# Effect of Pre-Milking Teat Foam Disinfection on the Prevention of New Mastitis Rates in Early Lactation

**DOI:** 10.3390/ani11092582

**Published:** 2021-09-03

**Authors:** Sarah Rose Fitzpatrick, Mary Garvey, Jim Flynn, Bernadette O’Brien, David Gleeson

**Affiliations:** 1 Teagasc, Animal and Grassland Research and Innovation Centre, Moorepark, Fermoy, P61 P302 Cork, Ireland; sarahfitzpatrick26@gmail.com (S.R.F.); Jim.Flynn@teagasc.ie (J.F.); Bernadette.OBrien@teagasc.ie (B.O.); 2 Department of Life Science, Institute of Technology Sligo, F91 YW50 Sligo, Ireland; Garvey.Mary@itsligo.ie

**Keywords:** mastitis, pre-milking teat disinfection, somatic cell count, foam teat disinfectant, dairy cows

## Abstract

**Simple Summary:**

The benefits of pre-milking teat disinfection have varied depending on management, practices and bacterial strains present in the environment, with some studies stating a reduction in the incidence of new infections and other studies stating little benefit of pre-milking teat disinfection. Furthermore, the effectiveness of pre-milking teat disinfection using foam has not previously been evaluated in a pasture-based dairy herd. This study has shown little benefit of applying a foaming pre-milking teat disinfectant in early lactation in a pasture-based dairy herd. However, the foaming teat disinfectant reduced bacterial counts on teat skin and may reduce the bacterial contamination of milk.

**Abstract:**

The objective of this study was to determine the benefit of pre-milking teat foam disinfection on the prevention of new infections by contagious and environmental bacteria in two spring calving herds managed outdoors (Herd 1 [H1]; 331 cows and Herd 2 [H2]; 142 cows). Four pre-milking teat preparation treatments were applied post calving; with each herd receiving two treatments; using a split udder design (for approx. 15 weeks). These treatments included; (1) ‘water wash, foam application and dry wipe (WFD) in H1′; (2) ‘water wash and dry wipe (WD)’ in H1; (3) ‘foam application and dry wipe (FD)’ in H2; (4) ‘no teat cleaning preparation (NP)’ in H2. Individual quarter foremilk samples were collected on four occasions and all clinical and sub-clinical cases were recorded. The mean SCC of quarter foremilk samples was 134 × 10^3^ cells/mL and 127 × 10^3^ cells/mL for WD and WFD, respectively, and 109 × 10^3^ cells/mL and 89 × 10^3^ cells/mL for NP and FD, respectively (*p* > 0.05). Lower bacterial counts were observed on teat skin that received a foaming treatment. Pre-milking teat disinfection using a foaming product may be of little benefit, in early lactation, for a pasture-based dairy herd.

## 1. Introduction

Post-milking teat disinfection has been proven an effective measure to reduce IMI within a dairy herd [1,2,3]. Pre-milking teat disinfection and cleaning is essential to reduce risk of exposing the open teat end to environmental pathogens [4] and is an important step in the production of high-quality milk [5,6]. Soiled teats can be an important source of contamination and depending on the effectiveness of pre-milking teat preparation, bacterial counts of teat skin may be increased, which may impact on bulk tank milk quality [5,6]. Exposure of teats to environmental bacteria can occur while cows are lying down or during their movement to the parlour [6], however, this exposure can be reduced by keeping cow areas clean and dry between milkings [4]. Additionally, a relationship was demonstrated between the rate of new IMIs and an increase of bacterial contamination on the teat skin and teat end [7]. Various studies have shown that the bacterial load on the teat skin surface can be influenced by the pre-milking teat preparation applied [8,9,10]. The uptake of pre-milking teat disinfection on farms has been low with only 2% of farms in New South Wales, Australia [11] and 14% of farms in Ireland [12] currently practicing pre-milking teat disinfection. This may be a consequence of the uncertain benefits and associated high labour costs [11], due to the number of cleaning steps required such as; cleaning teats, fore-stripping, applying a teat disinfectant product for at least 30 s and drying each teat thoroughly before cluster attachment [13,14].

Furthermore, there is significant evidence that the benefit of pre-milking teat disinfection is variable, with some studies stating a reduction in the incidence of clinical mastitis and IMI [4,15,16,17] and other studies stating no additional benefit of pre-milking teat disinfection when used in conjunction with post-milking disinfection [14,18,19]. Although, various pre-milking cleaning regimes have been shown to reduce bacterial numbers on the teat skin surface [6,9,20], the difference in results for pre-milking teat disinfection may possibly differ due to a variation in management practices between studies conducted in the United States, New Zealand, Australia, and Ireland (indoor housing vs. pasture-grazed). Bacterial strains identified in milk samples can also vary due to location. Studies carried out in New Zealand and Australia observed *Streptococcus uberis* and *Escherichia coli* as the most common pathogen isolated [11,18,19]. Whereas a study within an Irish dairy herd, staphylococcal isolates were the most prominent bacterial isolate on teat skin swabs (accounting for 49% of isolates) [21]. Furthermore, *Staphylococcus aureus* was shown to be the most predominate bacteria in clinical mastitis cases [22]. This agrees with a more recent Irish study where *Staph. aureus* was the most common pathogen isolated in clinical and sub-clinical quarter foremilk mastitis samples [14].

Pre-milking teat disinfectant can be applied as a spray, using a dip cup, or using a foaming gun or special foam-dipping cup. The use of a foaming pre-milking teat disinfectant may have additional benefits such as; allowing focus on the teats and not spraying the udder surface, it can reduce product usage rates as less volume is used, and foam clinging aids in the removal of dirt from the teats. Non-ionic and anionic surfactants within foaming products removing the surface tension on soiled teats, which may help to remove dirt quickly and efficiently. A post-milking foaming product was recently compared to a post-milking powdered chlorhexidine teat disinfectant, where the foaming product had less risk of IMIs caused by coagulase negative bacteria compared to the powdered product [23]. However, there is little knowledge on the effect of using teat disinfectant foam as a pre-milking cleaning regime in reducing mastitis rates and bacterial count on teat skin surface. While previous studies, undertaken in Ireland, did not observe a benefit of pre-milking teat disinfection over a full lactation [14], the use of pre-milking foam teat disinfection in early lactation may have a benefit against incidences of clinical and IMIs in the short term when incidence levels tend to be the highest.

Therefore, the objective of this study was to assess the benefits of applying foam to teats as a pre-milking teat treatment in early lactation in conjunction with post-milking teat disinfection, on reducing new mastitis levels in the short term and any possible impact on infections for a period after treatment had ceased.

## 2. Materials and Methods

This study was undertaken (under the approval of the Teagasc Animal Ethics Committee (ref. TAEC168-2017)) on two spring-calving Teagasc research herds (Herd 1 [H1], 311 cows and Herd 2 [H2], 142 cows) between January and July 2019. These herds were chosen as they were within a close proximity to the Teagasc research centre, which allowed for the collection and analysis of samples. The mean herd parity was 2.8 and 3.5 for Herds 1 and 2, respectively. Mean calving dates were 14th and 16th February for Herds 1 and 2, respectively. The pre-milking teat disinfectant used was Keno™ pure (lactic acid, CID Lines NV); which was diluted for use, following manufacturer recommendations. This product was applied as foam using the Cotswold™ Pure Foamer Foaming Gun, which is vacuum operated. The post-milking teat disinfectant “Deosan teatfoam” (chlorhexidine, polyhexamethylene, Johnson Diversey) was applied as a spray.

The study commenced on 15th January as cows calved and entered the parlour for the first milking. Cows were milked twice daily at approximately 7:00 a.m. and 2:30 p.m. in a 30 unit and an 18-unit swing-over side-by-side parlour (Dairymaster, Causeway, Co. Kerry, Ireland) in Herds 1 and 2, respectively. Four pre-milking teat preparation treatments were applied in a split udder design experiment, with each herd receiving two treatments. These treatments included; (1) ‘a water wash, foam application and dry wipe (WFD) in H1′; (2) ‘water wash and dry wipe (WD)’ in H1; (3) ‘foam application and dry wipe (FD)’ in H2; (4) ‘no teat cleaning preparation (NP)’ in H2.

The treatments were applied over a 15-week period with an extra sample point at 24 weeks after study commencement to determine any carryover effect of the pre-milking cleaning treatments. In Herd 1, the left front (LF) and left hind (LH) teats of all cows received a water wash (with running water), foam application and dry wipe, approximately 30 s after disinfection using disposable paper towels, before cluster attachment (WFD). The right front (RF) and right hind (RH) teats received a water wash (with running water) and were dry wiped with disposable paper towels before cluster attachment (WD). In Herd 2, the left front (LF) and left hind (LH) teats of all cows received foam disinfectant application and were dry wiped approximately 30 s after disinfection with disposable paper towels, before cluster attachment (FD). The disinfectant was applied to teats without any pre-cleaning of teats. The right front (RF) and right hind (RH) teats of cows in Herd 2 received no teat cleaning preparation treatment (NP). Teats were washed and dried with paper towels if teats were presented with a hygiene score of 4 [24].

Post-milking teat disinfectant was applied as a spray to all cows in each herd during the study. A high standard of cow and environmental hygiene was maintained throughout the study. Collecting yards and parlour approach yards were cleaned twice daily and roadways were maintained in good condition. Experienced full-time milking staff were employed for both herds, with two milkers required in herd 1 and one milker required in herd 2. Cow tails were clipped post calving. Individual cows in both herds were managed at pasture within two weeks of calving. After the experimental treatments ceased (week 15 (78 days in milk)), teat preparation reverted to the routines which were used on the respective farms during the previous year. All cow teats in Herd 1 were washed with running water, had a pre-milking teat disinfectant spray (“Deosan teatfoam” (chlorhexidine, polyhexamethylene, Johnson Diversey)) applied and were dry wiped with disposable paper towels prior to cluster attachment. No teat preparation was performed on teats of Herd 2 prior to cluster attachment but teats with a hygiene score of 4 were washed.

### 2.1. Quarter Milk Sampling Procedure

Individual quarter foremilk milk samples were taken in an aseptic manner on 4 occasions during early to mid-lactation: post-calving (4 days post-calving; sample 1), two weeks after first sample (18 days post-calving; sample 2), May (average days in milk (DIM) = 78; Sample 3) and July (average DIM = 138; Sample 4). Samples taken at days 4 and 18 were collected individually depending on the calving date of the cow, whereas samples taken at days 78 and 138 were collected in groups and all cows were sampled regardless of calving date. Quarter foremilk samples were obtained by trained personnel. Gloves were worn by personnel at all times and washed or disinfected between each cow. Before sample collection, each teat was scrubbed with cotton wool soaked in methylated spirits. Quarters were cleaned from front to rear. The first three squirts of milk were discarded to remove contaminated milk and material from the tip of the teat. Samples were taken from rear to front to avoid teat skin contamination. Samples were collected in sterile capped 30-mL bottles colour coded to represent each quarter. All quarter foremilk samples hada somatic cell count (SCC) quantified using a Bentley Somacount 300 (Bentley Instruments Inc., Chaska, MN). Quarter foremilk samples were cultured to isolate and identify bacteria using blood agar plates. The blood agar plate was divided into four equal sections and clearly identified with cow identification and quarter. Samples were plated using 10 μL aseptic loops, incubated at 37 °C, and examined after 18 to 24 h for growth morphologic features such as colony size, shape, colour, and haemolytic characteristics. For the duration of the study, individual cow bulk milk SCC was measured once weekly on a morning milk sample using a Fossomatic FC (Foss, Hillerød, Denmark). Individual cows with a bulk milk SCC ≥ 1,000,000 cells/mL were then quarter foremilk sampled to identify the infected quarter.

A critical infection value of 300,000 cells/mL was set for SCC to indicate the potential for sub-clinical mastitis in an individual quarter foremilk sample [25,26]. Quarter foremilk samples with SCC greater than the critical value on both day 4 and again on day 18 were considered infected at calve down and the quarter were excluded from the data set. Any quarters, which became clinically infected, were sampled before antibiotic treatment was applied and cultured to determine the bacteria present. A quarter was determined to be clinically infected if the milk was visibly abnormal (visible clots of milk or discoloured milk) or if the quarters had signs of inflammation (discoloured, swollen, warm or tender quarters/udders). Quarters treated for clinical mastitis during the trial were recorded and excluded from the SCC data set. Quarter foremilk samples with a SCC > 300,000 cells/mL with or without the presence of bacteria in two consecutive samples from a 10 μL milk sample were considered sub-clinically infected. Quarter foremilk samples with more than two bacterial species present were considered contaminated samples and were discarded and a quarter foremilk sample was retaken. Sub-clinical infections, which subsequently became clinical were no longer reported as sub-clinical and were excluded from the SCC data set but retained as a clinical infection.

### 2.2. Teat Swabbing Procedure

Teats of randomly selected cows (*n* = 20) in each herd were swabbed after the teat treatment and before cluster attachment on two occasions (average DIM of 34 and 62) to establish bacterial load on the teat skin. Twenty cows were chosen as a subset of herd size based on a previous study by Gleeson et. al. [14]. All teats from the selected cows were swabbed using two sterile swabs (Copan Italia S.p.A Via F. Perotti, 10 25,125 Bresica—Italy) moistened in sterile tryptic soy broth (TSB) (Merck Millipore, Cork, Ireland). One swab was used for the two teats receiving foaming treatments and one swab was used for teats receiving no foaming treatments in both herds. Swab samples were collected approximately 30 s after the teat cleaning treatment. Cows in both research herds were sampled on the same day at the morning milking and all swab samples were taken by the same person. A total of 160 swab samples were collected during the trial (40 cows × 2 swabs/cow × 2 days).

Swabs were drawn across the teat orifice and down the side of each teat avoiding contact with the udder hair. Immediately after sampling, swabs were placed into individual sterile bottles containing 10 mL of sterile TSB. Samples were placed in ice at 4 °C while being transported to the laboratory.

Swab samples were analysed for total bacteria count (TBC) within 1 h of sample collection and before dilution, samples were vortexed twice for 10 s. Following this, sterile tubes of maximum recovery diluent (Sigma-Aldrich, Ireland) were used to make 1:100 serial dilutions of the samples. One mL of this dilution was inoculated onto Petrifilm Total Aerobic Count plates (3M St. Paul, MN, USA) in triplicate, incubated for 48 h at 32 °C and then counted using the Petrifilm Plate Reader (3M). Swab samples were then frozen at −20 °C until analysed for staphylococcal isolates, streptococcal isolates, and coliform isolates within 14 days. For the bacterial isolate counts, samples were defrosted at room temperature and were left undiluted. Subsequently, 100 µL of each sample was plated, in triplicate, onto three separate agars; Baird parker agar (Merck Millipore, Burlington, MA, USA) for staphylococcal isolates, modified Edwards agar (Sigma-Aldrich, Saint louis, MO, USA) with 5% sterile blood for streptococcal isolates and MacConkey agar (Merck Millipore, Burlington, MA, USA) for coliform isolates. Following incubation at 37 °C for 24 h, microbial counts for each bacterial group were manually counted. Bacterial species within each isolate group were not defined. On both sampling days, a water sample was collected randomly to determine the TBC of the water used to wash teats during the trial.

### 2.3. Teat Skin Condition Scoring

Teat barrel skin condition was characterised using a modified version of the criteria established by [27,28]. Teat barrel skin condition was scored on a scale of 0 to 4, where 0 = teat skin is smooth and free from scales, cracks or chapping; 1 = teat skin shows some evidence of scaling with small cracks; 2 = teat skin is chapped and cracked; 3 = teat skin is severely damaged and ulcerative with scabs, open lesions or bloody appearance to cracks with redness indicating that inflammation is present; and 4 = teat skin has been subjected to physical injury (i.e., stepped on) not related to the treatment or the quarter is non-lactating. Teats were scored on two occasions (4 DIM and 138 DIM) by the same operator in both herds during the trial. The operator, using a lamp to illuminate the teat barrel, scored all teats immediately after cluster removal at the morning milking. A total skin condition score for each cow on inspection was obtained by calculating the average score of the two teats per treatment. The average score for each treatment was calculated by averaging the score for each set of teats.

### 2.4. Statistical Analysis

All data analysis was carried out using SAS 9.4. Counts were transformed using a log base-10 function. The data were heavily censored at the limit of quantitation, and to overcome distributional issues the main response for analysis was a difference of log values (between treatments, between front and hind quarters). Each herd was analysed separately during all statistical analysis in this study. The Mixed procedure was used to fit the analysis model for these differences and residual checks were made to ensure that the assumptions of the analysis were met. Covariance models were used to accommodate the repeated measures over time. Mean values on the log difference scale were back-transformed as required to ratios, on the count scale along with their confidence limits and mean SCC (× 10^3^ cells/mL) and presented as geometric mean SCC in the results.

Fisher’s Exact Test was used to test for independence in tables of bacterial type versus treatment or quarter, and logistic regression was used to fit analysis models for quarters with a SCC < 30,000 cells/mL and for quarters with a SCC > 200,000 cells/mL (bonus payments available at a SCC < 200,000 cells/mL). These groups were labeled ‘class 1 (SCC < 30,000 cells/mL) or ‘class 2 (SCC > 200,000 cells/mL)’. This was carried out with the Glimmix procedure in SAS with sampling day as a random effect, using the following equation:

*class1or2* = *Treatment*.

In examining time trends, the high level of censoring was a difficulty and the counts were categorised into above and at or below the level of quantitation (200,000 cells/mL). A trend over time was then fitted using logistic regression (Logistic procedure) to regress on days in milk using the following equation:

*LogDifferenceinTreatment* = *DIM*.

Bacterial counts (cfu/mL) on teat skin swab samples were transformed to base-10 logarithm for analysis. PROC GLIMMIX was used to perform multiple pair-wise comparisons. The LSMEANS statement in PROC GLIMMIX was used to differentiate statistical differences. The Log10 total bacterial count (TBC) for each teat swab collected was analysed using the following equation;

*Log_10_bacterialcount* = *Treatment* + *Day* + *Day*
*X*
*treatment*,

where treatment was the pre-milking teat preparation regime and day was the date of sampling. The Log10 bacterial count for each pre-milking teat preparation treatment (*n* = 4) were analysed separately within the herd (*n* = 2) using the same model. The Log10 bacterial counts of staphylococcal, streptococcal, and coliform isolates were analysed separately within each herd using the following equation;

*Log_10_bacterialcount* = *Treatment* + *Day* + *Bacteria* + *Treatment*
*X*
*Bacteria* + *Day*
*X*
*Bacteria*,

where treatment was the pre-milking teat preparation regime, day was the date of sampling and bacteria was the three bacterial isolate groups. The cow was the experimental unit when analysing TBC, staphylococcal, streptococcal, and coliform isolates. Residual checks were made to ensure assumptions of analysis were met.

## 3. Results

### 3.1. Quarter Foremilk Sample Results

A total of 311 cows (1244 quarters) in Herd 1 were allocated to the WD (622 quarters) and WFD (622 quarters) treatment groups and 142 cows in Herd 2 were allocated to NP (284 quarters) and FD (284 quarters) treatment groups. In Herd 1, one cow death, unrelated to the current study, occurred resulting in 310 cows in Herd 1. Eleven quarters were removed from analysis of both WD and WFD groups because they were considered clinically infected at calving. A further 29 and 28 quarters were removed from the groups WD and WFD, respectively, because they experienced a SCC above the critical value on both 4 and 18 DIM. Furthermore, seven and six quarters were removed from the WD and WFD treatment groups, respectively, due to milking ceasing during the study period in those teats. In Herd 2, six cows were culled, unrelated to the current study (e.g., lameness), resulting in 136 cows in Herd 2. Three and two quarters were removed from NP and FD treatments, respectively, due to a clinical case within 4 days of calving. A further 11 and 25 quarters were removed from the groups NP and FD, respectively, because they experienced a SCC above the critical value on both 4 and 18 DIM. Furthermore, eight and nine quarters were removed from the NP and FD treatment groups, respectively, due to milking ceasing during the study period in those teats.

**Figure 1:**
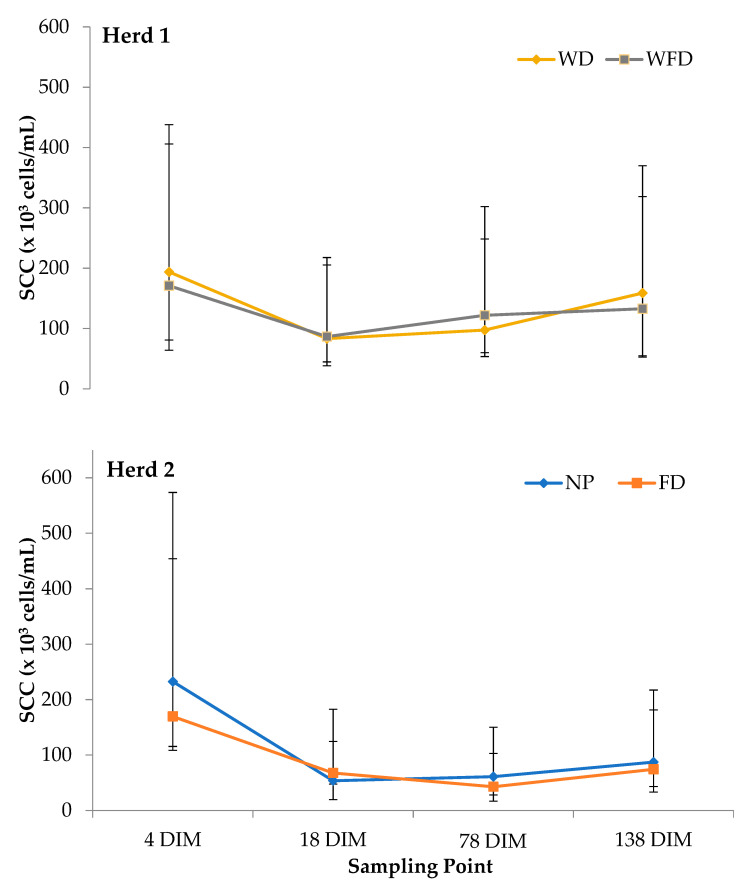
Geometeric mean somatic cell count (SCC) (× 10^3^ cells/mL) for four pre-milking teat disinfectant treatments on two herds (Herd 1: WD = wash and dry wipe and WFD = wash, foam application and dry wipe; Herd 2: NP = no teat preparation and FD = foam appliaction and dry wipe) at four sampling points (4 DIM, 18 DIM, 78 DIM, 138 DIM) during the study. Error bars indicate lower and upper 95% confidence intervals.

The geometric mean SCC for quarters which received four pre-milking teat preparation treatments at four sampling points in two herds can be observed in Figure 1. The mean SCC for Herd 1 was 134 × 10^3^ cells/mL and 127 × 10^3^ cells/mL for WD and WFD treatments, respectively. The mean SCC on Herd 2 was 109 × 10^3^ cells/mL and 89 × 10^3^ cells/mL for NP and FD treatments, respectively. A significant interaction of DIM on SCC of each individual quarter within the study was observed in both Herds 1 and 2, showing that SCC increased as DIM increased (*p* < 0.001). Within the study, there was no effect of lactation number/parity observed when comparing treatments within both herds. No sample date by treatment interaction was observed in both Herd 1 (WD and WFD) and Herd 2 (NP and FD). However, there tended to be a difference in SCC for Herd 1 between treatment groups WD (195 × 10^3^ cells/mL) and WFD (172 × 10^3^ cells/mL at 4 DIM; WD and WFD treatment difference ratio: 1.20 [0.99–1.45]) (*p* = 0.06). There also tended to be a difference between treatment groups in Herd 2 at 4 DIM (NP: 235 × 10^3^ cells/mL, FD; 171 × 10^3^ cells/mL; NP and FD treatment difference ratio: 0.75 (0.56–1.01)) (*p* = 0.06) and at 18 DIM (NP: 56 × 10^3^ cells/mL, FD; 70 × 10^3^ cells/mL; NP and FD treatment difference ratio: 0.77 (0.56–1.04)) (*p* = 0.08) (Table 1).

No benefit of pre-milking teat disinfection in early lactation was observed in terms of SCC within the study. In Herd 1, the mean SCC for each treatment group increased numerically between 78 DIM (cease of treatments) and 138 DIM by 61 × 10^3^ cells/mL to 159 × 10^3^ cells/mL for quarters previously receiving the WD treatment and by 11 × 10^3^ cells/mL to 133 × 10^3^ cells/mL for quarters previously receiving the WFD treatment. An increase in SCC was also observed in Herd 2. In NP treatment, SCC increased by 26 × 10^3^ cells/mL to 87 × 10^3^ cells/mL with quarters in the FD treatment increasing by 31 × 10^3^ cells/mL to 74 × 10^3^ cells/mL between 78 DIM and 138 DIM.

The proportion of quarters with SCC within the following categories; <30,000 cells/mL, between 30,000 and 100,000 cells/mL and ≥200,000 cells/mL, across 4 sampling points, are presented in Table 2. The proportions of quarters within the stated categories were similar across all treatments within each herd. A high proportion of quarters in the study had a SCC of <30,000 cells/mL, which was significantly higher than the proportion of quarters with a SCC of ≥200,000 cells/mL for treatments on both herds (*p* < 0.001). Furthermore, the proportion of quarters with a SCC of <200,000 cells/mL was; 0.89 and 0.91 for the WD and WFD treatments, respectively, in Herd 1, and 0.89 and 0.92 for NP and FD treatments, respectively, in Herd 2 (extracted from Table 2).

There was no significant difference between treatments for clinical and sub-clinical infections throughout the study for both Herds 1 and 2. Within the WD treatment, 14 (RF = 5; RH = 9) cases of clinical mastitis were recorded in comparison to 11 (LF = 3; LH = 8) clinical cases in the WFD treatment quarters in Herd 1. Additionally, 1 clinical case was recorded in the FD treatment group, with no cases of clinical mastitis recorded in the NP treatment group in Herd 2.

There was a similar number of sub-clinical infections in the WD treatment (*n* = 33 (RF = 16; RH = 17)) compared to the WFD treatments (*n* = 30 (LF = 10; LH = 20)) but a higher number of sub-clinical infections in the NP treatment (*n* = 17 (RF = 12; RH = 5)) than the FD treatment (*n* = 8 (LF = 4; LH = 4)). When herd size is considered. the lowest proportion of quarters infected was observed with the FD treatment (0.04) as compared to the NP treatment (0.07) in Herd 2. The WD treatment had numerically the highest proportion of infections (0.84) in Herd 1. The most commonly isolated bacteria from all quarter samples in Herd 1 were *Staph. aureus* (0.23) and *Strep. uberis* (0.02). In Herd 2, *Staph. aureus* (0.19) was proportionally the most prominent bacteria isolated from all quarter samples cultured (*n* = 1634), followed by *Streptococcus dysagalactiae* (0.006) (Table 3). A large proportion of all quarter samples analysed yielded no bacterial growth. (WD = 0.75, WFD = 0.72, NP = 0.78, FD = 0.83), with these proportions being smaller for the clinical and sub-clinical samples. Quarters, which became sub-clinically infected during the study continued to receive the teat treatment they were originally allocated. No benefit of applying a foaming pre-milking teat disinfectant to high SCC cows (quarters which exceeded the critical value of 300,000 cells/mL) was observed when compared to a wash and dry wipe or no teat preparation treatment (data not shown). Furthermore, in Herd 2, parity was found to have a significant treatment effect for the number of sub-clinically infected quarters (*p* < 0.002), which suggests as lactation number increased, SCC increased—regardless of treatment.

### 3.2. Teat Skin Swab Results

**Figure 2:**
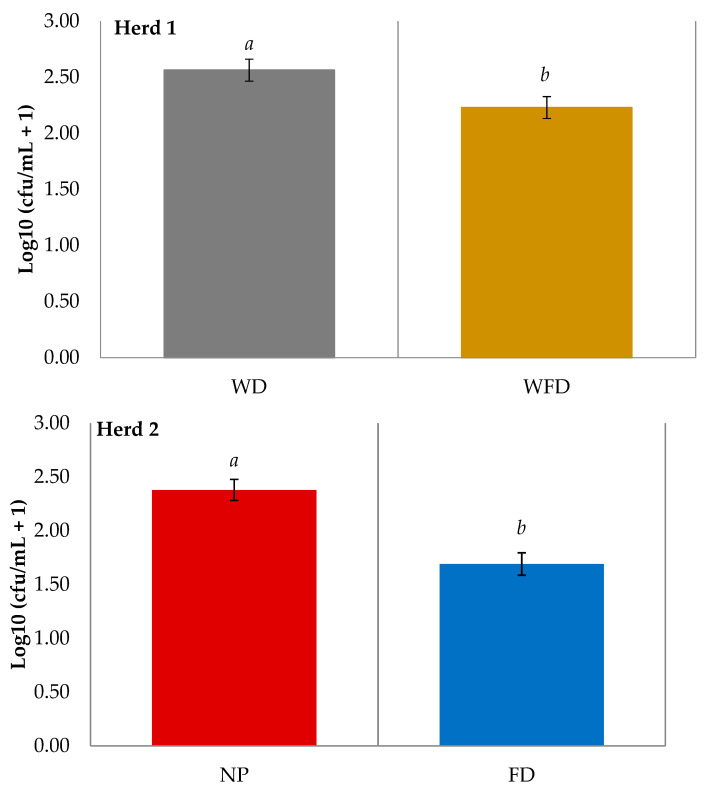
LS-means of the Log10 total bacterial count (TBC) on teat swabs after four different pre-milking teat preparation regimes across two dairy herds. (WD = wash with water and dry wipe, WFD = wash with water, foam application, and dry wipe, NP = no teat preparation, FD = foam application and dry wipe). *^ab^* Means with different letters differ significantly. Error bars indicate SEM.

**Figure 3:**
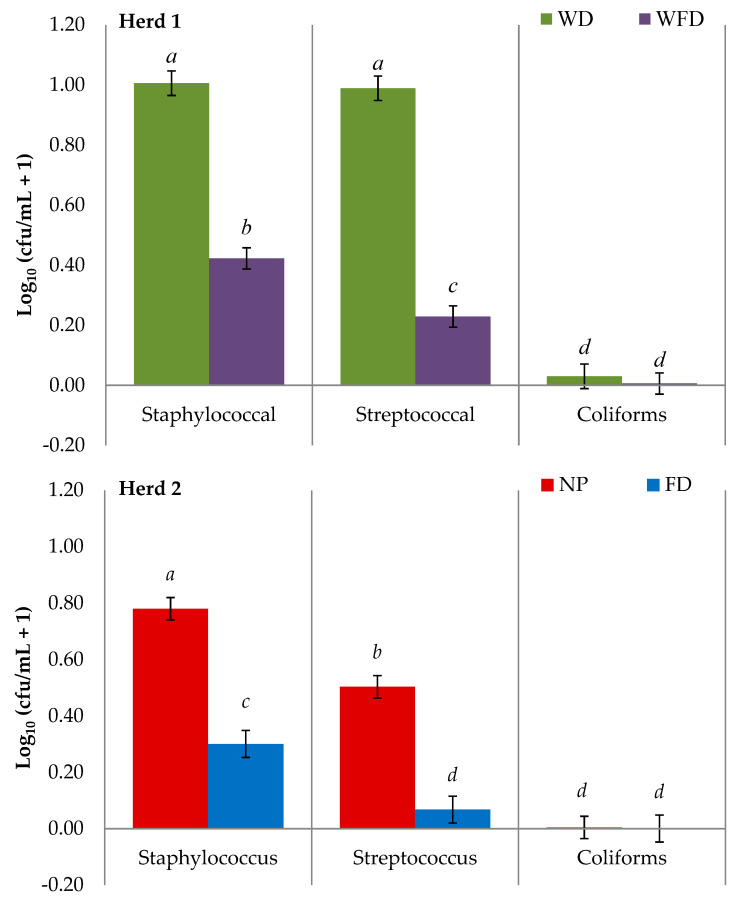
LS-means of the Log_10_ bacterial counts of staphylococcal, streptococcal, and coliform isolates on teat swabs taken after four different pre-milking teat preparation regimes across two dairy herds. (WD = wash with water and dry wipe, WFD = wash with water, foam application and dry wipe, NP = no teat preparation, FD = foam application and dry wipe). (*^a–d^*) Means with different letters within herd differ significantly. Error bars indicate SEM.

For teat swabs collected during the study, bacteria levels on the teat skin varied across each sample day. However, for TBC, staphylococcal, streptococcal, and coliform isolates there was no treatment by day effect, indicating that sampling day had no impact on treatment effectiveness. Staphylococcal isolates were the most prominent isolates recovered on teat skin swab samples (H1: WD = 0.48, WFD = 0.69; H2: NP = 0.59, FD = 0.83), followed by streptococcal (H1: WD = 0.52, WFD = 0.31; H2: NP = 0.41, FD = 0.16), and coliform isolates (H1: WD = 0.03, WFD = 0.03; H2: NP = 0.01, FD = 0.01), for both herds. Staphylococcal and streptococcal isolate counts were significantly higher for Herd 1 compared to Herd 2 (*p* < 0.001). In both herds, TBC (Figure 2) and both staphylococcal and streptococcal isolate bacterial counts (Figure 3) were significantly lower on teats treated with a foam disinfectant compared to teats which did not receive a foam treatment prior to cluster application (*p* < 0.001).

On the days where teat swab samples were collected, a water sample was collected randomly from wash hoses in the parlour (Herd 1 only). The water used to wash teats in Herd 1 had an average TBC of 2.38 log units, indicating that water quality should not have impacted on the treatments which included a wash with running water.

No significant differences were observed in teat condition scores between treatments WD and WFD in Herd 1 and NP and FD in Herd 2.

## 4. Discussion

The benefit of using a foaming product as a pre-milking teat disinfectant on quarter foremilk sample SCC is that new infection rates and bacterial counts on teat skin in early lactation can be determined. Results from this study show that the application of a foaming pre-milking teat disinfectant resulted in a numerically lower proportion (0.30) of infections as compared to no teat preparation and a lower proportion (0.11) when compared to wash and dry only in Herd 1. However, foam treatment had no significant impact on individual quarter SCC, when compared to a wash and dry (H1) or no teat preparation (H2). This result is similar to previous studies, which measured the benefit of pre-milking teat disinfection in pasture-based herds. Studies in Australia [11] and New Zealand [18] found that pre-milking disinfection is unlikely to reduce the incidence of clinical mastitis or new infection rates. Additionally, [18] found that pre-milking teat disinfection, in addition to post-milking disinfection, did not reduce SCC. The results of this current study also agree with a previous teat disinfection study conducted in Ireland where no benefit of pre-milking teat disinfection, in addition to post-milking disinfection, was observed when teats were sprayed with two different teat disinfectant products [14]. However, studies by [15,16,17] found pre-milking teat disinfection to be effective against new infections caused by *Str. uberis* and *E. coli* in dairy herds which were housed indoors.

The majority of clinical and sub-clinical cases in the current study were predominately associated with *Staph. aureus,* which is similar to the findings of a previous study in Irish dairy herds that observed that the greatest number of infections were caused by *Staph. aureus* [14]. *Staph. aureus* was also a prominent bacteria found in clinical and sub-clinical quarter foremilk samples in pasture-based herds in Australia and New Zealand [11,18]. During the current study, seven cows (Herd 1 = 6, Herd 2 = 1) had sub-clinical infections in both disinfected and non-disinfected quarters. This may be a negative aspect of the split udder design where an infected quarter could cross infect the neighbouring quarter [29].

Within the current study, a high proportion of quarters in all treatments had a SCC below 200,000 cells/mL. This figure may have been skewed as a number of quarters from each treatment, such as clinical quarters, were removed, possibly impacting on the high proportion of quarters falling into the low SCC category detailed above. The International Dairy Federation (IDF) has recommended that a SCC above 200,000 cells/mL may suggest that a quarter is infected [30], with previous studies showing that a SCC above 100,000 cells/mL may also imply an infection [31,32]. This further suggests that infection levels were low in both Herds 1 and 2. A low herd parity may also have contributed to the high proportion of quarters having a SCC lower than 200,000 cells/mL in this current study. However, parity had no impact on the pre-milking treatments on Herd 1 (mean parity 2.8) and Herd 2 (mean parity 3.5), which may be due to the low mean parity in each herd. A previous study performed on the data taken from dairy cows on farms within each province of Ireland has shown that as parity increases, SCC increases, with the lowest SCC being in the second parity [33]. While all treatments had increased SCC levels on day 138 as compared to day 78, the lowest increase in SCC was observed with FD (10%) as compared to an approximately 0.40 increase for the other three treatments during this period. The FD treatment also had numerically the lowest SCC on days 78 and 138.

Although the use of pre-milking teat disinfection may be of limited benefit in a pasture-based dairy system from a mastitis/infection point of view, it may help to improve the quality of milk as bacterial contamination is reduced through the cleaning of teats [6,20] and in particular when teats are heavily soiled [11]. This can be observed in studies where cows are housed indoors in free stalls, with various bedding substances, which found that pre-milking teat disinfection, along with post-milking disinfection, significantly lowered clinical mastitis and new IMIs compared to post-milking disinfection alone [16,17]. Furthermore, milking speed may have been increased through extra stimulation of the teat with an effective pre-milking teat-cleaning regime [34].

Within the current study, TBC, staphylococcal, streptococcal, and coliform isolate counts were lower on teat skin that received a foaming treatment compared to teats that received no foaming treatment prior to cluster attachment. This agrees with previous studies, which demonstrated that the application of a teat disinfectant can reduce bacterial levels on teat skin surface [6,9,14,20,21]. Although a relationship between bacterial load and the incidence of new infections was observed [7], the reduction in bacterial counts on the teat skin in this study did not lower the incidence of new infections.

Teat skin condition score did not differ between treatments within each herd in the current study. While teat skin condition may be expected to have improved for teats which received a foaming treatment (H1 = WFD, H2 = FD), due to teats being cleaned with a disinfectant containing teat skin conditioners (emollients), no differences were observed. During the study period, the foaming teat disinfectant product was applied using the Cotswold™ Pure Foamer Foaming Gun. This apparatus allowed for fast and effective application of the foam to teats. Due to vacuum operation, there was no requirement to re-fill teat dips cups during and/or after each milking.

## 5. Conclusions

Pre-milking teat disinfection using a foaming product may be of little benefit, in early lactation in reducing somatic cell counts, for a pasture-based dairy herd where there is a high standard of cow and environment cleanliness and herd parity is low. However, the use of pre-milking teat disinfection was found to reduce bacterial levels on the teat skin surface.

## Figures and Tables

**Table 1:** Treatment difference ratios of log values (95% confidence limits) for the difference in somatic cell count (SCC) of four pre-milking teat treatments (WD = wash and dry wipe; WFD = wash, foam application and dry wipe; NP = no teat preparation; FD = foam application and dry wipe) for two herds (herd 1 and 2) at four sampling points during the study.

Herd 1	WD vs. WFD	*p*-Value	Herd 2	NP vs. FD	*p*-Value
4 DIM	1.20 (0.99–1.45)	0.06	4 DIM	0.75 (0.73–0.56)	0.06
18 DIM	1.05 (0.85–1.30)	0.66	18 DIM	0.77 (0.56–1.04)	0.09
78 DIM	1.05 (0.79–1.40)	0.71	78 DIM	0.90 (0.60–1.35)	0.62
138 DIM	1.25 (0.90–1.73)	0.18	138 DIM	1.17 (0.73–1.87)	0.52

() parenthesis indicates the lower and upper 95% confidence limits.

**Table 2:** The proportion of quarters (number of quarters) from two herds (H1 and H2) for four pre-milking teat treatments (H1: WD and WFD, H2: NP and FD) with a somatic cell count (SCC) within categories, <30 × 10^3^, 30–100 × 10^3^ and ≥200 × 10^3^ cells/mL, across four sampling points.

	<30 × 10^3^ Cell/mL		30–100 × 10^3^ Cells/mL		≥200 × 10^3^ Cells/mL	
**Herd 1 (H1)**						
**Treatment**	**WD**	**WFD**	**WD**	**WFD**	**WD**	**WFD**
4 DIM	0.49	0.53	0.31	0.30	0.13	0.10
18 DIM	0.79	0.80	0.11	0.10	0.07	0.07
78 DIM	0.86	0.87	0.04	0.02	0.07	0.08
138 DIM	0.80	0.83	0.03	0.01	0.13	0.12
**Herd 2 (H2)**						
**Treatment**	**NP**	**FD**	**NP**	**FD**	**NP**	**FD**
4 DIM	0.52	0.58	0.24	0.26	0.16	0.12
18 DIM	0.80	0.82	0.08	0.09	0.06	0.06
78 DIM	0.84	0.83	0.03	0.03	0.08	0.06
138 DIM	0.86	0.86	0.01	0.01	0.10	0.09

WD = a water wash (with running water) and dry wipe. WFD = a water wash (with running water), foam disinfectant application and dry wipe. NP = no teat cleaning preparation. FD = foam disinfectant application and dry wipe. No. of quarters in each treatment: WD = 573, WFD = 575, NP = 250 and FD = 236.

**Table 3:** Bacteriological results of all quarter samples (including clinical and subclinical samples) for four pre-milking teat treatments on two herds (Herd 1 = WD (a water wash and dry wipe) and WFD (a water wash, foam application and dry wipe); Herd 2 = NP (no teat preparation) and FD (foam appliaction and dry wipe)).

	Treatment (Total No. of Samples)			
Organism	WD (573)	WFD (575)	NP (250)	FD (236)
	Herd 1		Herd 2	
*Staphylococcus aureus* (aB)	114 (0.20)	142 (0.25)	48 (0.19)	43 (0.18)
*Streptococcus uberis*	9 (0.02)	7 (0.01)	0 (0)	0 (0)
*Streptococcus dysagalactiae*	1 (0.002)	2 (0.003)	1 (0.004)	2 (0.008)
*Escherichia coli* (NH)	1 (0.002)	0 (0)	1 (0.004)	0 (0)
*Escherichia coli* (H)	1 (0.002)	0 (0)	0 (0)	0 (0)
Unclassified Gram (-) Cocci	20 (0.04)	20 (0.05)	5 (0.02)	5 (0.02)
No growth	431 (0.75)	415 (0.72)	195 (0.78)	195 (0.83)
Total	573	575	250	236

() Parenthesis indicates proportion of samples. aB = Beta-Haemolytic. H = Haemolytic. NH = Non-Haemolytic. (-) = Negative.

## Data Availability

None of the data is deposited in an official repository. Data can be available upon reasonable request.
